# Definition of the Gene Content of the Human Genome: The Need for Deep Experimental Verification

**DOI:** 10.1002/cfg.81

**Published:** 2001-06

**Authors:** Andrew J. G. Simpson, Sandro J. de Souza, Anamaria A. Camargo, Ricardo R. Brentani

**Affiliations:** The Ludwig Institute for Cancer Research Rua Professor Antônio Prudente 109, 4th floor, São Paulo SP 01509-010 Brazil

## Abstract

Based on the analysis of the drafts of the human genome sequence, it is being speculated that our species may possess an unexpectedly low number of genes. The quality of the drafts, the impossibility of accurate gene prediction and the lack of sufficient transcript sequence data, however, render such speculations very premature. The complexity of human gene structure requires additional and extensive experimental verification of transcripts that may result in major revisions of these early estimates of the number of human genes.


**Comparative and Functional Genomics**
*Comp Funct Genom* 2001; **2**: 169–175.
**DOI**: 10.1002/cfg.81

**Review Article**

### Definition of the gene content of the human genome: the need for deep experimental verification

Andrew J. G. Simpson*, Sandro J. de Souza, Anamaria A. Camargo and Ricardo R. Brentani
*The Ludwig Institute for Cancer Research, Rua Professor Antônio Prudente, 109, 4th floor, São Paulo, 01509-010, SP, Brazil*

*Correspondence to: A. J. G. Simpson, The Ludwig Institute for Cancer Research, Rua Professor Antonio Prudente, 109, 4th floor, São Paulo, 01509-010, SP, Brazil. E-mail: asimpson@node1.com.br*

Received: 4 April 2001
Accepted: 5 April 2001

### Abstract

**Based on the analysis of the drafts of the human genome sequence, it is being speculated that our species may possess an unexpectedly low number of genes. The quality of the drafts, the impossibility of accurate gene prediction and the lack of sufficient transcript sequence data, however, render such speculations very premature. The complexity of human gene structure requires additional and extensive experimental verification of transcripts that may result in major revisions of these early estimates of the number of human genes. Copyright © 2001 John Wiley & Sons, Ltd.**

**Keywords:** human genome; genes; DNA sequence; gene prediction; transcriptome; ESTs

### Introduction

Of all the justifications to sequence the human genome, the identification of the complete set of human genes is probably the most compelling. Certainly, it is the gene content, which is the facet of the genome, that is of the widest interest to both academic scientists and corporate research organizations alike. In this regard, there have been strong statements made about the gene content of the human genome, particularly in the press, following the completion of the draft human genome sequence. The general trend has been to draw attention to the conclusion that the human genome contains a surprisingly small number of genes that is not significantly removed from the number of genes present in the genome of lower eukaryotes that have been sequenced. The published manuscripts [18,29] describing this milestone in the evolution of science are somewhat more cautious, however, and the truth is that at the present time we have no real idea of the number of human genes, let alone what they encode and how they function. It is ironic that the essential completion of the human genome sequencing with the enormous investments of time and money that this has entailed, has not led to the most eagerly awaited portion of the information that it contains, the identification of human genes. The reason for this is that, although the human genome sequence is essential for the accurate description and cataloging of human genes, it is not sufficient. Human genes are highly complex structures and as yet we are not able to predict their presence with any certainty by inspection of genomic DNA sequence. Rather this absolutely requires direct experimental evidence in the form of transcript sequencing.

### Identification of genes within prokaryotic and eukaryotic genome sequences

The paradigm of whole genome sequencing as a route to determine gene complement has proved robust in the context of prokaryotic organisms [6,12]. Bacterial genomes are highly compact suggesting a strong selective pressure to reduce genome size. Thus, genes are head to tail with one another and, crucially for gene hunters, uninterrupted by introns. Thus the standard procedure for gene identification, is to first identify open reading frames with an algorithm such as Glimmer [8]. Subsequently these ORFs are annotated, or assigned putative function,

Copyright © 2001 John Wiley & Sons, Ltd.

on the basis of comparison with known genes or proteins from other organisms using programs such as BLAST [2].

Even for bacterial genomes this is not fool-proof, however, as an arbitrary lower limit to the size of ORFs taken as real has to be imposed close to those that can be expected to occur by chance within non-coding DNA sequences. This is a serious limitation in the absence of any similarity between the putative ORF and known genes and proteins. Nevertheless, the identification of approximately one gene per kb of genome sequence has been possible for all bacteria for which the genome sequence is publicly available. The confidence level is high due to the combined evidence of long open reading frames and similarity with previously defined genes. Thus, although there may be some error in the precise definition of the initiation codon, in general there is no need for further confirmation of gene structure by transcript sequencing or microarray experimentation. In addition, it should be remembered that this kind of gene identification is based on complete, high quality sequences that contain no gaps and where all ambiguities have been resolved.

There are two fundamental differences between prokaryotic and eukaryotic gene structure that complicate the identification of eukaryotic genes within genome sequence. The first is that the relative proportion of the genome occupied by genes is considerably smaller in eukaryotes. Although there is approximately one gene per kb in bacterial genomes, there is only about one per 100 kb in the human genome [18,29]. Thus we are dealing with a structure where the genes are two orders of magnitude more widely spread. Far more importantly, however, is that eukaryotic genes are fragmented into exons separated by intervening introns. Thus the first step in gene identification, that of putative ORF detection, is not possible in the context of the human genome. This is the fundamental problem of human gene identification based on genomic sequence alone. Indeed, this problem is more acute in the human genome than in the other eukaryotic genomes sequenced due to the significantly greater sizes of human introns [18,29].

By aligning previously sequenced, complete cDNAs with human genomic sequence the general characteristics of human gene structure have been outlined. The comparison of these data with other eukaryotic genomes shows that the average overall length of coding regions in *C. elegans* (the worm), *D. melanogaster* (the fly) and for *H. sapiens* (human) is 1311, 1497 and 1340 bp respectively [18]. In addition, it reveals that in all three organisms internal exons are generally between 50 and 200 bp with the average exon sizes for the worm and for human being 218 bp and 147 bp respectively. On the other hand, intron sizes are found to be significantly larger in human. In the worm and the fly the averages are 267 bp and 487 bp respectively while in human the average is roughly ten times greater, 3300 bp [18]. Moreover, the variation in intron size is markedly greater in human. This highly dispersed and variable nature of human genes makes them simply impossible to detect with any accuracy by simple inspection of the human genome, even aided by the most sophisticated algorithms produced to date. Thus, when all is said and done, the sequencing of the human genome did not lead to gene identification, as was the expectation. Genes previously defined by cDNA sequencing could be aligned to the genome, allowing their precise mapping and the definition of intron-exon structure, but no new genes could be identified from genome sequence alone.

### Gene identification in the human genome drafts

Although gene discovery did not feature in the completion of the draft genome sequence, both the International Human Genome Sequencing Consortium (IHGSC) and Celera projects catalogued the position of those genes that are already well defined by comparison of high quality, full-length mRNA sequences with the draft genome [18,29]. This allowed the intron-exon boundaries of the corresponding genes to be defined for the fist time in many cases. Importantly, however, this exercise also permitted the suitability of the draft genome for novel gene identification to be assessed.

In both projects the RefSeq database [22] was used as the source of high quality full-length transcript sequences. RefSeq is a carefully, manually curated, non-redundant data set that contains most genes for which a reliable full-length mRNA sequence is available [22]. At the time of the genome annotation, RefSeq contained 10 271 human mRNAs. When these were compared to the IHGSC draft it was found that of the RefSeq

Copyright © 2001 John Wiley & Sons, Ltd. 

sequences, 92% showed high stringency alignment over at least some portion of their length, 85% could be aligned over at least half of their length but in only 52% could an essentially complete alignment be achieved [18]. Thus almost half of known genes were only found to be partially represented in the genome sequence demonstrating the rudimentary nature of the draft genome sequence and hence its lack of present suitability as a basis for novel gene identification.

Even if it were possible to accurately predict genes based on sequence data alone, the draft at the time of publication is arguably simply too fragmented to make this a worthwhile exercise. Indeed, on examining the largest 10 genes in Ref Seq, Aach *et al.*, found that only six had both ends in the same contig of the human genome assembly, two genes had ends in different contigs and the remaining two had only one end that could be found within the genome sequence [1].

In the case of the Celera [29] sequence it was possible to identify only 6538 of the genes corresponding to the RefSeq sequences on the basis of a match against the genome for at least 50% of their length with >92% identity. Again, this rather small number reveals the extent of fragmentation and error in the sequence and the difficulty therefore of using it for novel gene prediction.

Thus in both cases we have to take the highly fragmented nature of the draft sequences into account when assessing estimates of gene numbers. Clearly over the coming months an essentially finished sequence will become available that will circumvent this problem. The question then remains as to how to identify genes in this high quality sequence.

### Human gene prediction

Despite the shortcomings of the draft sequence as a source for gene discovery, efforts were made by the IHGSC in this direction by building what they term an initial gene index (IGI) [18]. This was produced firstly by using the Ensembl system that involves a prediction program together with confirmatory evidence from ESTs, proteins, protein motifs and sequences from other organisms. In addition, a second approach was taken whereby attempts were made to extend EST and mRNA matches using statistical approaches. As a result of these studies a total of 31 778 protein predictions were made of which 14 882 represented known genes [18].

The limitations of this approach were assessed by comparison with newly discovered genes arising from independent work that were not used in the gene prediction effort. Of 31 such genes, only 19 (68%) were represented in the predictions. Furthermore, of each gene predicted an average of 79% was detected [18]. In a less direct, but larger scale approach a set of 15 294 full-length mouse cDNAs was examined and again only 69% showed any similarity with the predicted human genes [17,18]. Moreover, of 817 mouse hypothetical transcripts for which there were no corresponding human genes in RefSeq, Human Unigene or Ensemble database, only 174 perfectly matched GenScan predictions and 322 sequences did not hit any exons predicted by GenScan. The remaining 311 showed partial matches because GenScan did not predict one or more exons [17]. Although detailed calculations of sensitivity, fragmentation and prediction rates are tempting, the best conclusion is that these approaches are so inexact that there is little point in extrapolating from such theoretical exercises to the number, structure or function of human genes.

A combination of predictions and comparisons with proven transcripts were also utilized in order to identify human genes within the Celera draft [29]. In addition to the genes identified on the basis of RefSeq comparisons, a further 11 226 genes were predicted using a novel system named ‘Otto’ that attempts to reproduce in an automated way the kind of assessment of transcript evidence that a human annotator undertakes. In addition, 8619 genes detected on the basis of at least two confirmatory lines of evidence (ESTs, protein, mouse genome matches) for separate *de novo* gene predictions. This latter number increased to 21 350 if only one line of confirmatory evidence was taken as sufficient [29]. Thus the overall numbers that result from this analysis are very similar to those obtained from the IHGSC project [18]. Although, the kind of detailed assessment of the limitations of the predictions provided in the IHGSC paper was not presented, the numbers provided suggest that a similar level of accuracy and completeness is probable. Indeed, in this regard the data of Aach *et al.* conclude that the quality of the two draft sequences are of a similar quality as judged by sequence gaps, continuity, consistency between the

Copyright © 2001 John Wiley & Sons, Ltd.

two sequences and patterns of DNA-binding protein motifs [1].

Both studies leave us at a very preliminary stage as judged by the similarity of the numbers of genes found and the detailed assessment of the lack of accuracy of the methodology utilized as detailed in the case of the IHGSC manuscript. This lack of precision of prediction of human genes has been amply documented elsewhere and the data in the genome papers are entirely consistent with the overall position of this field [5,13].

### Estimates of the number of human genes

The final overall estimates of the number of genes in the human genome are 30–40 000 in the case of the IHGSC and 26–38 000 in the case of Celera [18,29]. These estimates were made despite the shortcomings outlined above. This does not mean that the estimates are wrong only that it is too early to be sure. They are consistent with extrapolations of gene numbers from the published chromosome 21 and 22 sequences [10,14]. In addition, the human genome papers quote recent independent estimates as supporting evidence for these low numbers. One of these papers involves the calculation of the gene number by comparing the number of known genes and ESTs and arrives at estimates of approximately 34 000 [11]. The known genes used were those for which we have a full-length mRNA or those annotated on chromosome 22. The estimate depends on these sets being representative of all genes particularly in terms of expression level. At least in terms of the full-length mRNAs this is clearly not the case and thus the assessment may be flawed. For example, if we take Unigene cluster size as a rough estimate of expression level, we can find 38 789 clusters in Unigene Build 128 composed of two to 10 sequences (representing rarely expressed genes) of which 1985 (5.1%) contain a full-length cDNAs whereas there are 4572 clusters of 100 or more sequences (representing highly expressed genes) of which 4249 (92.9%) contain a full-length cDNA (unpublished observations).

The other paper involves comparison between human and *Tetraodon nigroviridis* (a pufferfish) DNA as the basis of exon identification [7]. This estimate arrives at the similar number of 28–34 000 genes. Again, however, this estimate crucially relies on the relatedness of the fully characterized human genes and pufferfish sequences reflecting that of the yet to be defined human genes and pufferfish sequences. It should be noted that a companion paper of those cited above that simply depended on the very careful clustering of available EST sequences came to the conclusion that there are in the range of 120 000 human genes [21]. This paper did not have the benefit of the human genome sequence to aid clustering and may certainly have overestimated gene number due to the complexity of alternative splicing and polyadenylation. Nevertheless, it serves to show how essentially the same data can lead to very different conclusions when analyzed in different ways using different assumptions.

### The need for further transcript sequencing

In the closing sections of their paper the Celera team admit: 'As was true at the beginning of genome sequencing, ultimately it will be necessary to measure mRNA in specific cell types to demonstrate the presence of a gene' [29]. We wholeheartedly agree with this statement. A pervasive view is that the sequencing of the genome of other species may also be a strategy for gene identification [3,7]. Certainly, comparison with organisms at an appropriate evolutionary distance is a valuable way of identifying probable genes. The more genomes there are to compare the better such predictions will be. We believe, however, that this will never substitute for transcript sequencing due to the difficulty in identifying the exact start and stop of each exon not to mention the added value of alternative splicing and expression patterns that transcript sequencing provides.

That is not to say that transcript sequencing is not without its shortcomings. Firstly, it is clear that the amount of transcript data that will be required to find all human genes will be enormous. At the time of the annotation of the draft human sequences, around 10 000 putative full-length sequences were available and in the order of 3-million ESTs. This was clearly woefully deficient given the huge uncertainties in finding human genes that are alluded to above. It may ultimately be necessary to obtain the full sequence at least one example of every transcript in every cell type and all developmental stages to identify all genes (the

Copyright © 2001 John Wiley & Sons, Ltd. 

attraction of gene prediction is that these daunting requirements are circumvented). In addition, it will be necessary to cover each gene several times in different tissues in order to identify splicing alternatives that are often tissue specific. One approach to this multiple coverage is to adopt a high-throughput approach to transcript sequencing in a shotgun like format. This can now be effectively achieved using a combination of 3′ and 5′ EST sequencing together with our own Open Reading Frame EST (ORESTES) approach that tags the central portions of transcripts [9]. ORESTES is also a more realistic approach for searching for rare, tissue specific transcripts. The ORESTES methodology strongly normalizes and uses only minimal amounts of mRNA permitting such surveying to be contemplated. One could contemplate using ORESTES to provide the initial evidence of a transcript followed by a planned experimental strategy such as cDNA library screening or RACE to find the rest of the transcript. Alternative splices could then be sought by RT-PCR analyses.

The other principle problem with transcript sequencing is its technical difficulty that is significantly greater than that of genome sequencing particularly in relation to template preparation. In this regard trace amounts of genomic DNA are often incorporated into both ORESTES and conventional ESTs. Thus, careful analysis has to be undertaken and confirmatory evidence such as the presence of a splice site or the generation of the same putative transcript fragment from distinct libraries always sought.

We take the view that a combination of exhaustive transcript sequencing together with the availability of a high quality genome sequence is an absolute requirement of the compilation of a meaningful human gene catalogue. The first steps in this direction are now possible by careful and complete cross-analysis between the transcript and genomic sequence data. Such mapping exercises give an idea of the complexity of the situation and the necessity of an extensive investment in further experimental analysis. Figure 1 shows an example of where we have mapped all available transcript data to a region of the X-chromosome. The example shows three regions of clustered ESTs. We suspect that those in the middle comprise a gene since a putative full-length sequence has been generated. Such sequences have not been generated for the other two clusters however. At the present moment, based on careful analysis of the 3′-sequences and the likelihood that they represent authentic poly-A tails we predict that the left hand cluster corresponds to a single gene while that on the right actually represents two distinct, but closely positioned, genes.

Further in relation to the complexity of the relationship between transcript structure and genome sequence several situations well documented in the literature are pertinent. Firstly, there is the question of the generation of so-called anti-sense transcripts. It is well known that many genes are transcribed in both directions producing anti-sense transcripts that appear to play an important regulatory role [28]. However, there are many examples where these anti-sense transcripts also contain ORFs and are indeed transcribed [4,20,23,24,25]. These thus should be considered for all intents and purposes distinct genes that would be difficult ever to predict without transcript, and eventually, protein analysis. In addition there are intriguing examples of distinct genes being placed within the introns of other genes [15,16,19,27]. Again, this is a very difficult situation to predict without transcript data.

We are in the process of systematically closing EST clusters to generate full-length transcript sequences by making predictions from the genome mapping of the ESTs followed by RT-PCR experimentation. These is a powerful approach that permits the examination of the genome piece by piece and does not require the essentially chance generation of a full-length transcript from a cDNA library to confirm the structure of a gene. In the process of leading up to this project we have already compared the gene annotation on chromosome 22 with our own prediction of transcribed regions based on ORESTES sequences that have been generated in the FAPESP/LICR-Human Cancer Genome Project being currently concluded in Brazil [26]. This project has generated in excess of one-million human ESTs from the central regions of expressed human genes. When we mapped only those that correspond to human chromosome 22 from amongst our first 250 000 sequences we were able to identify based on stringent criteria a further 219 regions not described in the original annotation. We believe, but have not yet established, that these may correspond to more than 100 novel genes on this chromosome alone [26].

Copyright © 2001 John Wiley & Sons, Ltd. 

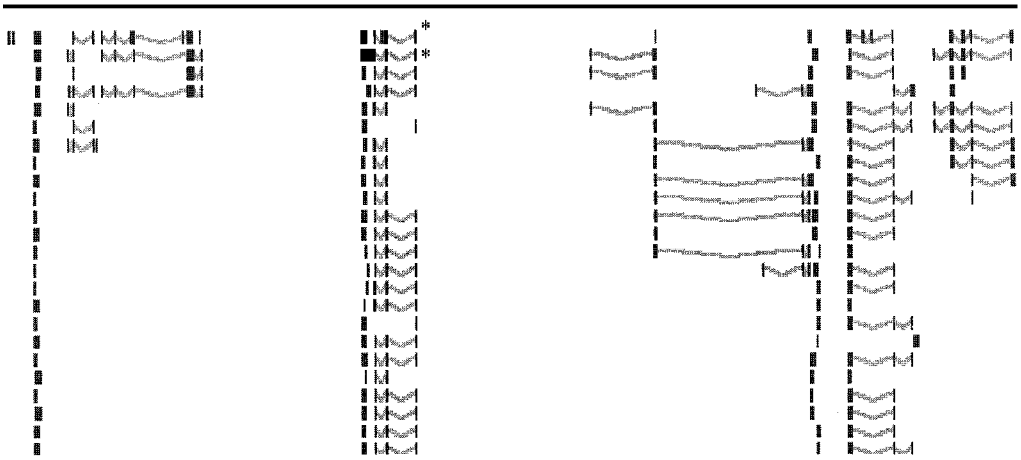

**Figure 1.** Alignment of ESTs (light bars) and cDNAs (dark bars with asterisks) to part of a BAC clone (acc.no.AC002477) from the human X chromosome. The "vs" between the vertical bars indicate that the linked exons are contained in the same experimentally derived transcript sequence. The long uppermost horizontal bar represents 114 kb of genomic DNA sequence

### Conclusion

Although we now have a draft human genome sequence and although the expressed gene content of the human genome is of fundamental interest and has attracted intense investigation, we are far from knowing what the gene content of the human genome is. It is possible that indeed we are only at the very beginning of our definition of this 'periodic table' of the human body. Any conclusions concerning our transcriptome or indeed proteome are very premature. This is not only due to the huge variety of transcripts that are generated from the genome due to alternative splicing and polyadenylation but also because we have yet to identify the genes themselves to any level of certainty. Although there is much talk of the proteome being the next step, it might be foolhardy to venture too far into this world without first establishing a very firm foothold in that of the transcriptome first.

### Acknowledgements

We thank the Fundação de Amparo a Pesquisa do Estado de São Paulo (FAPESP) and the Ludwig Institute for Cancer Research (LICR) for their generous support of our work.

### References

1. Aach J, Bulyk ML, Church GM, *et al.* 2001. Computational comparison of two draft sequences of the human genome. *Nature* **409**: 856–859.
2. Altschul SF, Madden TL, Schaffer AA, *et al.* 1997. Gapped BLAST and PSI-BLAST: a new generation of protein database search programs. *Nucleic Acids Res* **25**: 3389–3402.
3. Batzoglou S, Pachter L, Mesirov JP, Berger B, Lander ES. 2000. Human and mouse gene structure: Comparative analysis and application to exon prediction. *Genome Res* **10**: 950–958.
4. Borsu L, Presse F, Nahon JL. 2000. The AROM gene, spliced mRNAs encoding new DNA/RNA-binding proteins are transcribed from the opposite strand of the melanin-concentrating hormone in mammals. *J Biol Chem* **51**: 40576–40587.
5. Clarevie JM. 1997. Computational methods for the identification of genes in vertebrate genomic sequences. *Hum Mol Genet* **6**: 1735–1744.
6. Clayton RA, White O, Fraser CM. 1998. Findings emerging from complete microbial genome sequences. *Curr Opin Microbiol* **1**: 562–566.
7. Crollius HR, Jaillon O, Bernot A, *et al.* 2000. Estimate of human gene number provided by genome-wide analysis using *Tetraodon nigroviridis* DNA sequence. *Nat Genet* **25**: 235–238.
8. Delcher AL, Harmon D, Kasif S, White O, Salzberg SL. 1999. Improved microbial gene identification with GLIMMER. *Nucleic Acids Res* **17**: 4636–4641.
9. Dias-Neto E, *et al.* 2000. Shotgun sequencing of the human transcriptome with ORF expressed sequence tags. *Proc Natl Acad Sci U S A* **97**: 3491–3496.
10. Dunham I, Shimizu N, Roe BA, *et al.* 1999. The DNA sequence of human chromosome 22. *Nature* **402**: 489–495.
11. Ewing B, Green P. 2000. Analysis of expressed sequence tags indicates 35,000 human genes. *Nat Genet* **25**: 232–233.
12. Fraser CM, Eisen JA, Salzberg. 2000. Microbiol Genome sequencing. *Nature* **406**: 799–803.
13. Guigo R, Agaewal P, Abril JF, Burset M, Fickett JW. 2000. An assessment of gene prediction accuracy in large DNA sequences. *Genom Res* **10**: 1631–1642.
14. Hattori M, Fujiyama A, Taylor TD, *et al.* 2000. The DNA sequence of human chromosome 21. *Nature* **405**: 311–319.

Copyright © 2001 John Wiley & Sons, Ltd.

15. Herzog H, Darby K, Hort YJ, Shine J. 1996. Intron 17 of the human retinoblastoma susceptibility gene encodes an actively transcribed G protein-coupled receptor gene. *Genome Res* **6**: 858–861.
16. Kaufmann D, Gruener S, Braun F, *et al.* 1999. EVI2B, a gene lying in an intron of the neurofibromatosis type 1 (NF1) gene, is as the NF1 gene involved in differentiation of mrlanocytes and keratinocytes and is overexpressed in cells derived from NF1 neurofibromas. *DNA Cell Biol* **18**: 345–336.
17. Kawai T, Shinagawa A, Shibata K, *et al.* 2001. Functional annotation of a full-length mouse cDNA collection. *Nature* **409**: 685–690.
18. Lander ES, Linton LM, Birren B, *et al.* 2001. Initial sequencing and analysis of the human genome. *Nature* **409**: 860–921.
19. Levinson B, Kenwrick S, Lakich D, Hammonds JRG, Gitschier J. 1990. A transcribed gene in an intron of the human factor VIII gene. *Genomics* **7**: 1–11.
20. Li AW, Too CKL, Murphy PR. 1996. The basic fibroblast growth (FGF-2) antisense RNA (GFG) is translated into a mutT-related protein in vivo. *Biochem Bioph Res Comm* **223**: 19–23.
21. Liang F, Holt I, Pertea G, *et al.* 2000. Gene index analysis of the human genome estimates approximately 120,000 genes. *Nat Genet* **25**: 239–240.
22. Maglott DR, Katz KS, Sicotte H, Pruit KD. 2000. NCBI's LocusLink and RefSeq. *Nucleic Acids Res* **28**: 126–128.
23. Memes JP, Benzow KA, Koob MD. 2000. The SCA8 transcript is an antisense RNA to a brain-specific transcript encoding a novel actin-binding protein (KLHL1). *Hum Mol Genet* **9**: 1543–1551.
24. Miyajima N, Horiuchi R, Shibiya Y, *et al.* 1989. Two erbA homologs encoding proteins with different $T_3$ binding capacities are described from opposite DNA strands of the same genetic locus. *Cell* **57**: 31–39.
25. Rother KI, Clay OK, Bourquim JP, Silke J, Schaffner W. 1997. Long non-stop reading frames on the antisense strand of heat shock protein 70 genes and prion protein (Prp) genes are conserved species. *Biol Chem* **378**: 1521–1530.
26. de Souza SJ, Camargo AA, Briones MRS, *et al.* 2000. Identification of human chromosome 22 transcribed sequences with ORF expressed sequences tags. *PNAS* **97**: 12690–12693.
27. Valleix S, Jeanny JC, Elsevier S, *et al.* 1999. Expression of human F8B, a gene nested within the coagulation factor VIII gene, produces multiple eye defects and developmental alterations in chimeric and transgenic mice. *Hum Mol Genet* **8**: 1291–1301.
28. Vanhee-Brossolet C, Vaquero C. 1998. Do natural antisense transcripts make sense in eukaryotes? *Gene* **211**: 1–9.
29. Venter JC, Adams MD, Myers EW, *et al.* 2001. The sequence of the human genome. *Science* **291**: 1304–1351.

Copyright © 2001 John Wiley & Sons, Ltd. 
